# A multi-study analysis enables identification of potential microbial features associated with skin aging signs

**DOI:** 10.3389/fragi.2023.1304705

**Published:** 2024-01-11

**Authors:** Tyler Myers, Amina Bouslimani, Shi Huang, Shalisa T. Hansen, Cécile Clavaud, Anissa Azouaoui, Alban Ott, Audrey Gueniche, Charbel Bouez, Qian Zheng, Luc Aguilar, Rob Knight, Magali Moreau, Se Jin Song

**Affiliations:** ^1^ Center for Microbiome Innovation, Jacobs School of Engineering, University of California San Diego, La Jolla, CA, United States; ^2^ L’Oréal Research and Innovation, Clark, NJ, United States; ^3^ Department of Pediatrics, University of California San Diego, La Jolla, CA, United States; ^4^ L’Oréal Research and Innovation, Aulnay sous Bois, France; ^5^ L’Oréal Research and Innovation, Chevilly La Rue, France; ^6^ Department of Computer Science and Engineering, University of California San Diego, La Jolla, CA, United States; ^7^ Shu Chien-Gene Lay Department of Engineering, University of California San Diego, La Jolla, CA, United States; ^8^ Halıcıoğlu Data Science Institute, University of California San Diego, La Jolla, CA, United States

**Keywords:** microbiome, aging, face, multi-study investigation, wrinkles, TEWL (transepidermal water loss)

## Abstract

**Introduction:** During adulthood, the skin microbiota can be relatively stable if environmental conditions are also stable, yet physiological changes of the skin with age may affect the skin microbiome and its function. The microbiome is an important factor to consider in aging since it constitutes most of the genes that are expressed on the human body. However, severity of specific aging signs (one of the parameters used to measure “apparent” age) and skin surface quality (e.g., texture, hydration, pH, sebum, etc.) may not be indicative of chronological age. For example, older individuals can have young looking skin (young apparent age) and young individuals can be of older apparent age.

**Methods:** Here we aim to identify microbial taxa of interest associated to skin quality/aging signs using a multi-study analysis of 13 microbiome datasets consisting of 16S rRNA amplicon sequence data and paired skin clinical data from the face.

**Results:** We show that there is a negative relationship between microbiome diversity and transepidermal water loss, and a positive association between microbiome diversity and age. Aligned with a tight link between age and wrinkles, we report a global positive association between microbiome diversity and Crow’s feet wrinkles, but with this relationship varying significantly by sub-study. Finally, we identify taxa potentially associated with wrinkles, TEWL and corneometer measures.

**Discussion:** These findings represent a key step towards understanding the implication of the skin microbiota in skin aging signs.

## Highlights

• We confirm the positive link between chronological age and skin microbiome diversity, but we also observed a global positive association between microbiome diversity and grade of Crow’s feet wrinkles, one of the key signs of skin aging, although the relationship varied among the included sub-studies. We additionally observed a negative link between microbiome diversity and transepidermal water loss.
• The link between Crow’s feet wrinkles and microbial features were explored while considering the effect of individual studies and chronological age as a confounder, identifying several potential biomarkers.
• Building a multi-study analysis using independent studies is a valuable method to bolster sample sizes and address questions not possible by individual studies alone. However, as the sub-studies are often generated by different principal investigators and methods, analysis relies on data harmonization and the use of analytic tools that are able to account for those differences.

## 1 Introduction

As the most exposed organ to the external environment, the human skin harbors a highly diverse and individualized community of microorganisms that can vary considerably across different body sites (Costello et al., 2009; Oh et al., 2014) and over one’s lifetime. In fact, skin microbial composition has been shown to be more predictive of chronological age in adults than oral or gut microbial composition (Huang et al., 2020). This is no surprise given the many physiological changes that skin undergoes with age. The development of skin microbial colonization after birth, along with the maturation of the skin’s immune system ultimately contributes to the establishment of skin homeostasis in childhood (Capone et al., 2011). During puberty, around 10 years of age, increased secretion of sebum on the face promotes colonization of lipophilic bacteria, particularly *Cutibacterium acnes*, known to participate to some extent to acne (Dréno et al., 2018; McLaughlin et al., 2019) Studies have identified the dominance of this species in teens and young adults aged between 15 and 25 years (Mourelatos et al., 2007; Barnard et al., 2016). During adulthood, the skin microbiota can be relatively stable if environmental conditions are also stable (Oh et al., 2016). However, as people reach older age (55–60 years), studies have found that their face skin microbial communities shift with an increase in diversity (Jugé et al., 2018; Kim et al., 2019; Li et al., 2020; Wu et al., 2020; Ma et al., 2021; Howard et al., 2022), and a reduction in *C. acnes* reported to be associated with several surface parameters changes such as reduced sebum secretion (Shibagaki et al., 2017; Howard et al., 2022), and increased skin dryness (McGinley et al., 1975). These patterns appear to be accentuated among centenarians (Zhou et al., 2023a). While the number of studies associating skin microbiome changes with chronological age continues to grow, the microbiome link with indicators of skin aging and skin quality remains underexplored. This is an area important for consideration as the microbiome represents most of the genes that are present on the human body (estimated to be 100x the number of human genes (Gilbert et al., 2018), offering both greater genetic diversity and flexibility for manipulation than the human genome. Just as the gut microbiome is emerging as an important factor of healthy aging and even lifespan (Sonowal et al., 2017; Wilmanski et al., 2021), a more detailed view remains to be investigated to associate skin microbial signatures with youthful, healthy skin quality.

Skin aging is a natural and intrinsic process that involves shifts in endogenous processes (e.g., cellular metabolism, immune activity, hormones) combined with external environmental factors such as exposure to UV and pollution. Skin aging is characterized by a change in immune function and a decrease in sweat and sebum secretions, thus resulting in significant alterations in skin surface physiology including an increase in pH and alteration of the lipid composition (Cotterill et al., 1972; Pochi et al., 1979; Wilhelm et al., 1991; Farage et al., 2008; Howard et al., 2022). These physiological changes, in turn, may likely affect the skin microbiome. For example, functional skin microbiome studies have identified differentially abundant bacterial metabolism pathways between different age groups with a predominance of amino acid metabolism pathways in younger groups and amino acid degradation pathways in older groups (Kim et al., 2019).

External factors such as stress, tobacco use, diet, air pollution, and UV exposure account for 80% of the skin aging signs over time (Yaar, 2006; Poljšak et al., 2012; Flament et al., 2013; Marionnet et al., 2014; Rinnerthaler et al., 2015). Interestingly, these factors have also been reported to be linked to skin microbial composition and functional changes which in turn may be one mechanism by which skin quality is impacted. For example, in the context of air pollution, a shift in microbiome metabolic function was associated with signs of premature skin aging and dry skin (Leung et al., 2020).

Interestingly, although there is now evidence that the skin microbiome changes with chronological age, chronological age may not directly relate to “apparent age”, which is defined by parameters of skin aging signs and surface quality (e.g., texture, hydration, pH, sebum, etc.). For example, older individuals can have young looking skin (young apparent or perceived age) and young individuals can have more severe skin aging signs than the average population, thus presenting an older apparent age (Flament et al., 2021). Therefore, skin microbial markers of chronological age may not systematically overlap with those for apparent age.

A better understanding of the relationship between the skin microbiome and skin quality and aging is a key path to the development of new microbiome-based solutions to interfere in the skin aging process to improve skin quality and appearance as the populations age. Here, we present a framework for the identification of microbiome signatures associated with skin aging signs using a multi-study analysis of 13 microbiome and clinical datasets totaling more than 1000 subjects.

## 2 Materials and methods

### 2.1 Data description and study participants

Microbial sequence data and metadata (data about the samples and subjects) from 13 different observational cohort studies were aggregated into a multi-study analysis. A summary of the studies is provided in Table 1, describing the technical and cohort characteristics for each study.

**TABLE 1:** Summary characteristics of the 13 skin microbiome datasets included in the multi-study analysis.

Study ID	Age range	Country	Ethnicity	Collection date	# volunteers	Grade of wrinkles range	TEWL range	Corneometer range
1	55–69	Mauritius	Asian	Jun 2018	30	4–6	NA	NA
2	55–69	Mauritius	Asian	Jun 2018	32	4–6	NA	NA
3	55–65	Singapore	Asian	Sept 2018	27	NA	11.10–21.60	NA
4	55–65	France	Caucasian	Sept 2018	29	3–4	9.63–21.35	NA
5	56–65	Japan	Asian	Jan 2019	28	NA	7.75–30.49	NA
6	19–43	France	Caucasian	Nov 2017	26	NA	10.48–25.25	28.58–77.82
7	19–44	France	Caucasian	Jan 2018	32	NA	9.69–28.38	NA
8	18–45	France	Caucasian	Mar 2017	40	NA	9.42–31.40	34.40–83.80
9	19–44	France	Caucasian	Sept 2018	32	NA	NA	33.80–75.90
11^31^	25–45	China	Asian	Oct 2015	200	1–6	NA	24.80–79.60
12	20–55	United States	Not collected	Nov 2017	103	NA	7.40–24.20	21.26–75.97
13	30–45	United States	Not collected	Dec 2017	52	NA	4.03–25.10	49.36–75.07
14	33–50	France	Caucasian	Jun 2016	29	0–3	9.90–28.20	37.00–86.00

The subjects in the studies were all females between the ages of 18–70 years. They were recruited and sampled under similar protocols (Supplementary Table S1). These non-interventional studies were approved by the necessary Institutional Review Board or Research Ethics Committee, depending on the countries. Each study was conducted according to the principles expressed in the World Medical Association Declaration of Helsinki and national and EU regulations. All volunteers provided written informed consent prior to any study-related procedure. All participants provided information regarding health status, medical history, and daily habits. The participants were non-smokers, did not receive antibiotics or systemic antifungals at least 1 month prior to sampling, did not have acute cutaneous disorders, nor had used depigmenting/whitening or exfoliating products at least 1 month prior to sampling. To standardize the skin condition, the participants were asked to wash their face with a provided neutral soap without anti-bacterial compounds at least 1 day prior to sampling. Last, shampoo and soap were applied 48 and 24 h respectively before sampling. No other products were allowed on the face until sampling was performed. More details about the studies are available in Supplementary Table S1.

### 2.2 Skin sampling and measurements

Skin sampling was conducted as reported earlier (Leung et al., 2020). Briefly, microbiota sampling was conducted in a climate-controlled room at 21 ± 1°C and 60% humidity. The samples for microbiome analysis were collected by using sterile cotton-tipped dry swabs pre-moistened with a 0.15 M NaCl with 0.1% Tween 20 solution. Swabs were then rubbed firmly on the cheek for 60 s to cover a surface area of 2 cm^2^. After sampling, each cotton swab was placed into a cryotube and immediately flash-frozen in liquid nitrogen and stored at −80°C.

Skin quality was determined by three main measurements: grade of Crow’s feet wrinkles (GCFW), hydration, and transepidermal water loss (TEWL). GCFW was determined by clinical scoring the Crow’s feet wrinkles on a standardized 1-6 point scale as described earlier (Qiu et al., 2011; Flament et al., 2020), hydration of the upper epidermal layer was measured on the cheeks by a Corneometer^®^ CM825 (Courage and Khazaka Electronic) which measures the change in the dielectric constant due to skin surface hydration, and TEWL was measured on the cheeks with a Tewameter^®^ TM300 (Courage + Khazaka ElectronicGmbH), which measures water evaporation from the skin (Berardesca et al., 2020)

### 2.3 16S ribosomal RNA sequencing

Genomic DNA was extracted from the frozen swabs by using the PowerSoil DNA Isolation kit (MOBIO Laboratories Inc., Carlsbad, United States) according to the manufacturer’s instructions (Soares et al., 2016). For all studies, the variable region 1-3 of the 16S rRNA gene was amplified using previously described methods (Massiot et al., 2021). To prepare 16S amplicon libraries, 2.5 μL of DNA was used for a first PCR amplification step of 25 cycles with primers V1-27S and V3-535R using the KAPA HiFi HotStart ReadyMix PCR kit (Roche Diagnostics, Laval, Québec, Canada). After purification with Agencourt AMPure XP beads (Beckman Coulter, Mississauga, Ontario, Canada), a second PCR amplification step was performed to incorporate specific index adaptors for multiplexing. The quality of final libraries was examined on a TapeStation 2200 (Agilent Technologies, Santa Clara, CA, United States) and quantified using the Qubit 3.0 fluorometer and/or the Quant-iT dsDNA Assay (ThermoFisher Scientific, Canada). Subsequently, 16S libraries were pooled together in equimolar ratio and sequenced on an Illumina MiSeq system for 300bp paired-end sequencing at the Genomics Center, CHU de Québec-Université, Laval Research Center, Canada. Study 11 was sequenced using Illumina MiSeq system for 300bp paired-end sequencing at the School of Energy and Environment, City University of Hong Kong, Hong Kong SAR, China as previously described (Leung et al., 2020) (Table 1; Supplementary Table S1).

### 2.4 Data processing

Metadata variable names were standardized for the same fields that differed among studies (e.g., TEWL_right_face vs. TEWL_right_cheek), and those that contained values for more than 40% of samples were retained for further analysis. Sequence data from the 13 studies were processed consistently through the web tool Qiita (Gonzalez et al., 2018), which utilizes functions from the widely used microbiome data analysis tools Qiime (Caporaso et al., 2010) and Qiime2 (Bolyen et al., 2019). To standardize the input data for downstream analysis, sequences were trimmed to 250 nucleotides and put through a noise reduction process called deblurring (Amir et al., 2017). Taxonomy was assigned to each resulting unique amplicon sequence variant (ASV) using a classifier that was pre-trained on the Silva database (release 138) (Yilmaz et al., 2014). The resulting feature tables denoting read counts of each ASV for each sample, were combined, and reads that classified as “Mitochondria” and “Chloroplast” were filtered from the dataset prior to downstream analyses.

### 2.5 Descriptive analysis

Spearman correlation (confidence level = 0.95, alternative hypothesis = two sided) and linear mixed effects models were used to determine whether the metadata fields of interest (age, GCFW, TEWL, and corneometer) co-vary with one another. All descriptive analyses of the microbiome data were performed using Qiime2. Sequences were rarefied to 1000 reads per sample prior to any downstream analyses and included calculation of alpha diversity (Shannon), which captures the amount of microbial diversity present in each sample. Spearman correlation was used to show the direction and strength of the relationship between Shannon diversity and the metadata fields of interest in the collective dataset. A linear mixed effects model was utilized to account for study as a random variable. These statistical tests were performed using R version 4.1.2 and plotted using the R package ggplot2 (v.3.3.5).

Preliminary analysis of beta diversity (e.g., Unweighted/Weighted Unifrac), which captures differences between samples, indicated that there is a strong study effect, which we sought to reduce by collapsing ASVs to their assigned species. This resulted in 5,666 species-level features (down from 131,395 ASVs). We then assessed the amount of variation explained by factors of interest in the species-collapsed data using a permutational multivariate analysis of variance (PERMANOVA) (Anderson, 2001). Downstream analyses were conducted with the collapsed table of features.

### 2.6 Differential abundance analysis

In order to identify taxa associated with age and aging signs, we performed differential abundance analyses using BIRDMAn (Bayesian Inferential Regression for Differential Microbiome Analysis) (Rahman et al., 2023). Given the known study effects and age being highly correlated with GCFW, both study ID and age were used as covariates in the model. Additionally, feature tables were filtered to a prevalence of 15 across samples for model compilation. All models were run with the following parameters (num_iter = 200, num_warmup = 50, chains = 4).

BIRDMAn outputs two values of interest. First, the Gelman-Rubin diagnostic (R-hat) is provided as a metric to evaluate reliability of model differential estimates. R-hat compares the variance of Markov Chain Monte Carlo chains run in parallel and checks that the chains have converged on the same distribution. Second, the differentials show the log-fold change of individual features with respect to the variables included in the model (i.e., study, age, GCFW, TEWL, and corneometer), which are used to rank the features. Effectively, the top ranked features are those that show the highest log fold change with the changing variable, and thus are the most associated with, for example, higher GCFW. Diagnosis of the negative binomial models was completed using built in functions for chain convergence (R-hat), log pointwise predictive density, and posterior predictive checking.

### 2.7 Identifying microbial taxa of potential interest

Using the feature ranks output by BIRDMAn (Rahman et al., 2023), we iteratively selected the top n and bottom n features (taxa) and calculated the log-ratio of their abundances. We calculated log-ratios (i.e., n features in the numerator over n features in the denominator), instead of the log-fold change of individual features, to account for the compsitional nature of microbiome data (Morton et al., 2019). We then calculated the Spearman correlation of these log-ratios with the target variable (i.e., GCFW, TEWL, or corneometer), and determined the optimal combination of the top n and bottom n ranked taxa that maximized the correlation.

## 3 Results

### 3.1 Metadata harmonization

Standardization of metadata parameters across studies resulted in sample sizes of *n* = 988, 402, 551, and 621 for age, GCFW, TEWL, and corneometer respectively. TEWL and corneometer were relatively evenly distributed across studies (Supplementary Figure S1). Host age and GCFW showed uneven distributions, with some studies covering restricted ranges. However, the studies collectively covered a wide range of ages (18–70 years old), and GCFW (Supplementary Figure S1). As several of the studies included samples from both the right and left cheeks, samples were filtered to only include one sample per individual (left cheek for those with both samples) to avoid bias for statistical analyses. Data harmonization resulted in final sample sizes of *n* = 653, 314, 305, and 430 for age, GCFW, TEWL, and corneometer respectively.

### 3.2 Descriptive analysis of microbiome diversity and composition with skin parameters

Despite the localization of value ranges for host age and GCFW by study, when analyzed both collectively and by study, we found that host age was expectedly and significantly correlated with GCFW (Spearman correlation *R* = 0.58, *p* = 3.88E-29; linear mixed effects model *β* = 0.12, *p* = 1.8E-31). However, such a relationship was not detected between age and TEWL (Spearman correlation *R* = −0.09, *p* = 0.12; linear mixed effects model *β* = −0.04, *p* = 0.22) or corneometer measurements (Spearman correlation *R* = −0.03, *p* = 0.65; linear mixed effects model *β* = −0.05, *p* = 0.54).

**FIGURE 1:**
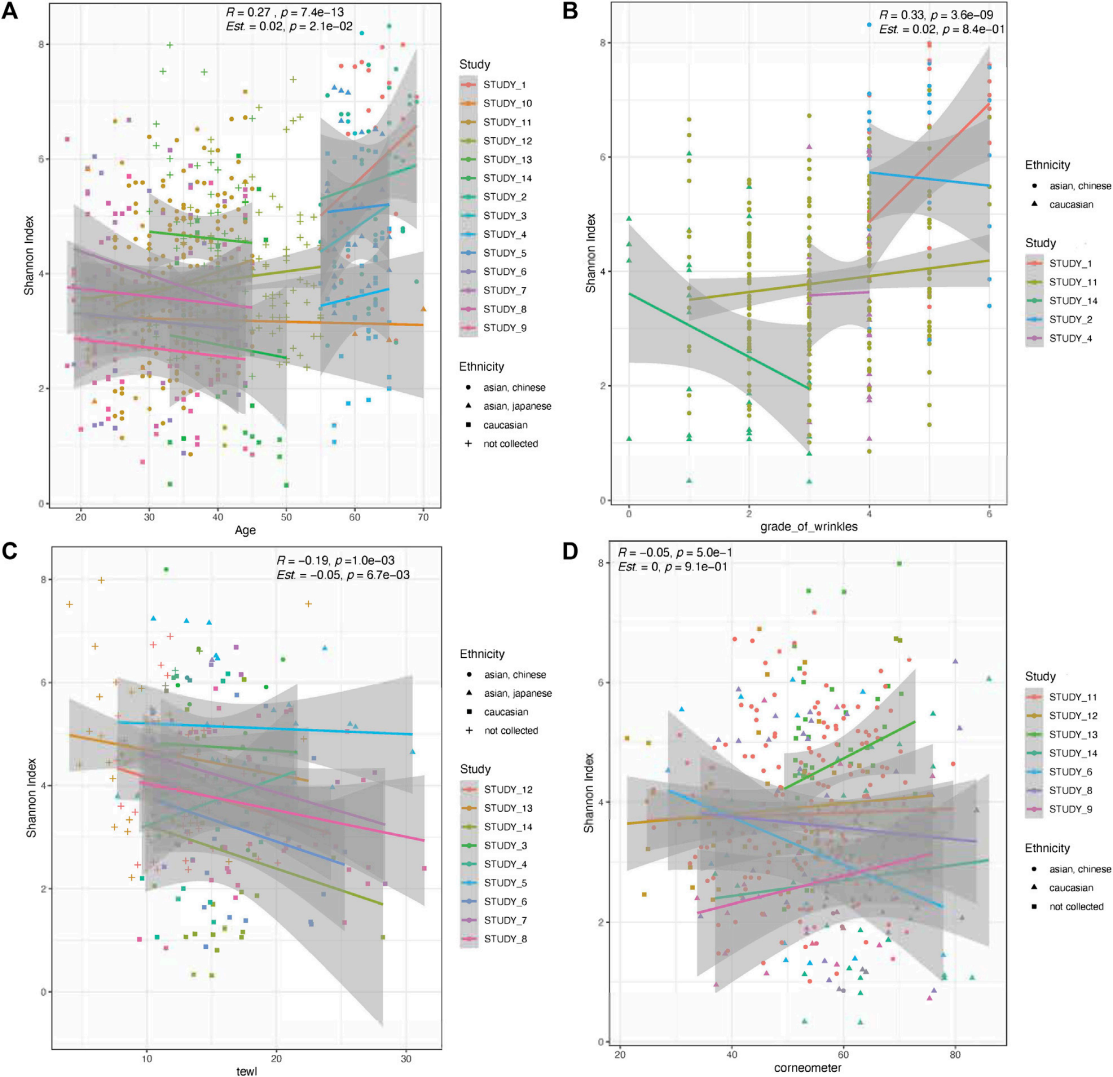
Microbial skin diversity correlations with age, grade of wrinkles, TEWL, and corneometer. Scatterplots show alpha diversity (Shannon’s index) among the groups with increasing age **(A)**, grade of wrinkles **(B)**, TEWL **(C)**, and corneometer **(D)**. Show in the inset are the Spearman correlation values (R, p) for the full dataset, along with the statistics for a mixed effects model [Est (β), p] using study as a random effect. Lines show a fitted regression line for each study, and shaded areas show the 95% CI. Point colors represent study and shapes represent ethnic groups.

Host age and GCFW were both positively correlated with microbial diversity (Shannon’s index) (*R* = 0.27, *p* = 7.4e-13; *R* = 0.33, *p* = 3.6e-09; Figures 1A, B) when using the dataset collectively without accounting for inter-study variation. When the study variable was included as a random effect, host age remained significantly and positively related to diversity (*β* = 0.02, *p* = 0.021), whereas GCFW did not (*β* = 0.02, *p* = 0.84). Of note, we observed a significant negative relationship between microbial diversity and TEWL (R = −0.19, *p* = 0.001; *β* = −0.05, *p* = 0.0067; Figure 1C). No significant relationship was detected between microbial diversity and corneometer values (R = −0.05, *p* = 0.5; *β* = 0, *p* = 0.91; Figure 1D).

Variation in microbiome composition was also significantly and most strongly affected by the study variable (PERMANOVA *R* ^2^ = 0.51, *p* = 0.001), followed by age (*R* ^2^ = 0.08, *p* = 0.001), GCFW (*R* ^2^ = 0.07, *p* = 0.001), and TEWL (*R* ^2^ = 0.06, *p* = 0.001). Corneometer did not significantly explain microbiome variability (*R* ^2^ = 0.01, *p* = 0.084).

### 3.3 Differential abundance of bacterial taxa associated with skin parameters

**FIGURE 2:**
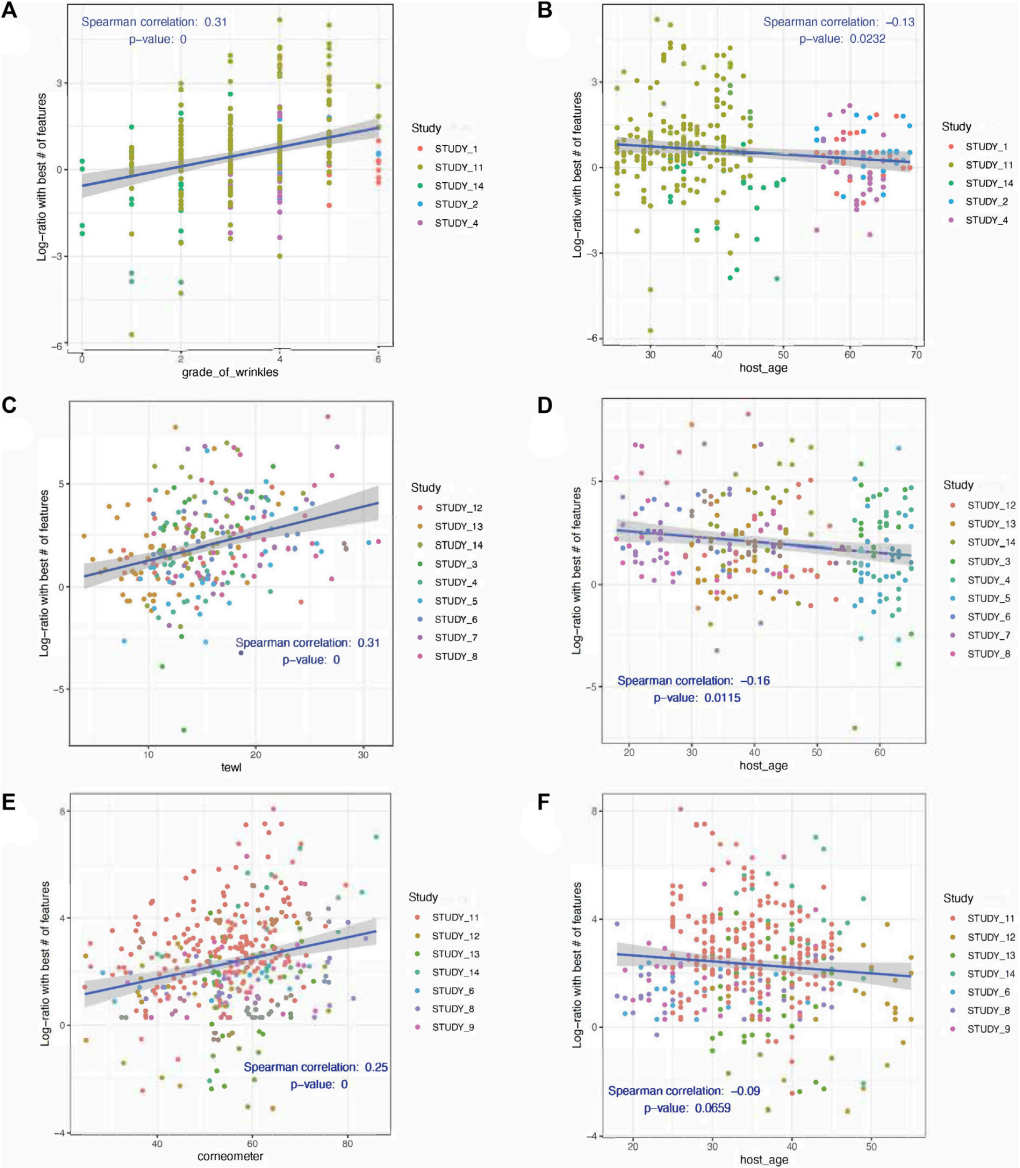
Plots showing the log ratios of the top n ranked taxa over the lowest n ranked taxa as identified by Birdman to be associated with **(A)** grade of wrinkles, **(C)** TEWL, and **(E)** corneometer. The number of taxa used in the log-ratios were those that maximized the correlation with grade of wrinkles (top 22 over the bottom 13 taxa), TEWL (top 1 over bottom 12), or corneometer (top 4 over bottom 14). Log-ratios are graphed as a function of the variables of interest as well as age for those same taxa **(B, D, E)**. The Spearman correlation values (R, p) are presented in each plot. Each point represents one sample, with colors indicating study.

**FIGURE 3:**
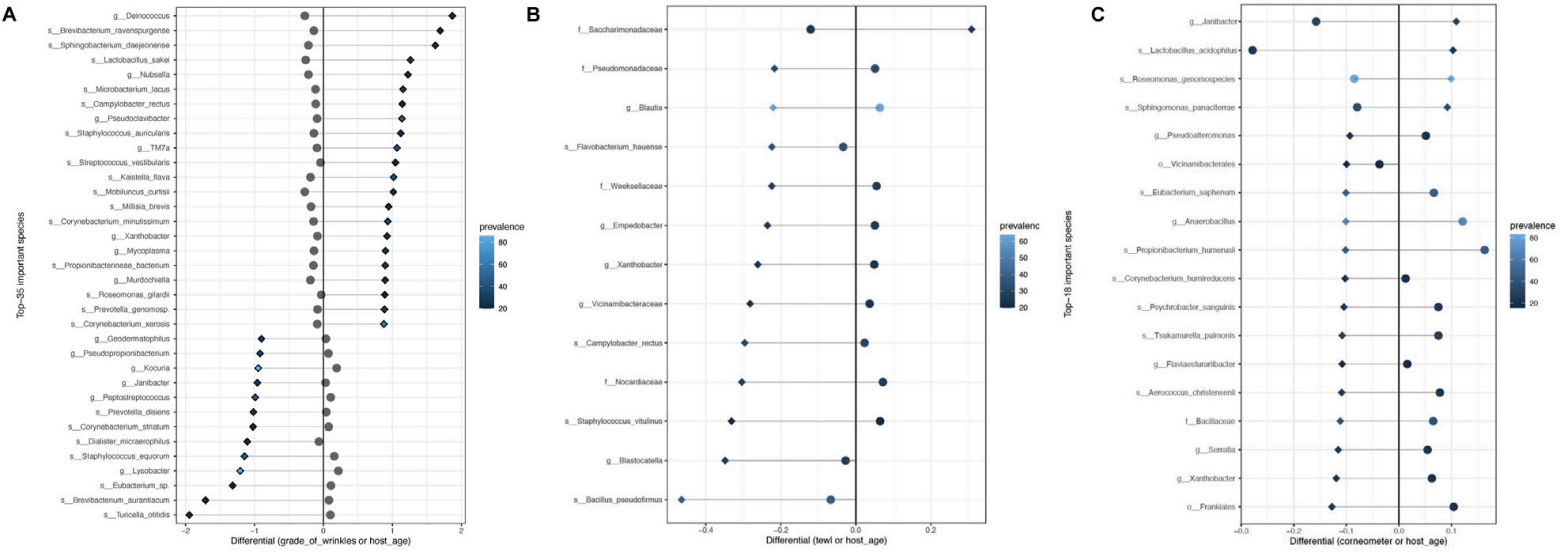
List of taxa most associated with **(A)** higher grade of wrinkles (top 22 listed) and lower grade of wrinkles (last 13 listed), **(B)** higher TEWL (top 1 listed) and lower TEWL (last 12 listed), and **(C)** higher corneometer (top 4 listed) and lower corneometer (last 14 listed), plotted with their differentials for the variable of interest (shown as diamonds) and age (shown in circles). Prevalence (number of samples in which the taxa is present) is shown by the color gradient.

Differential abundance analysis using BIRDMAn was conducted to identify taxa associated with age, GCFW score, and measurements of TEWL and corneometer. Including study ID and age as covariates in the models resulted in microbial taxa ranked by their association with the specific variable of interest. The full set of results from the BIRDMAn analysis can be found in Supplementary Tables S2–S5. First, the list of taxa was reduced to focus on those of highest potential importance, by analyzing log-ratios of the taxa most associated with higher values (highest ranked) against those most associated with lower values (lowest ranked) for each of the skin parameters. For GCFW, we found that a log ratio of the 22 highest ranked taxa against the 13 lowest ranked taxa maximized the correlation with GCFW (Figure 2A; *R* ^2^ = 0.31, *p* < 0.05). Plotting the centered-log-ratio for individual taxa allowed us to see their specific relationship with GCFW more clearly (Supplementary Figures S3–S6). While many of these taxa were at low prevalence, a few of the taxa were present in a larger number of samples, thus marking potential taxa of interest. We found that samples from skin with lower grades of wrinkles were associated with some commensal taxa such as the genera *Staphylococcus, Kocuria*, *Peptostreptococcus*, and *Lysobacter,* (Figure 3A). (Tanno et al., 2021; Lebeer et al., 2022; Zanchetta et al., 2022) Our results also identified environment-related bacteria including *Brevibacterium* and *Kaistella* that are often associated with skin alterations and inflammatory conditions such as psoriasis and senile xerosis (Huang et al., 2021; Li et al., 2021). These taxa were enriched in the samples from subjects with higher grades of wrinkles (Figure 3A). It should be noted that a second taxon assigned to *Brevibacterium* was identified as being associated with lower GCFW as well, suggesting effects on skin may be different depending on the strain, as previously reported for the skin commensal *S. epidermidis* (Landemaine et al., 2023). Meanwhile, although age and grade of wrinkles are highly related, the log-ratio of wrinkle-associated taxa did not show a similar positive correlation with age, but rather a significant negative relationship (Figure 2B; *R* ^2^ = −0.13, *p* = 0.0232).

A similar analysis, BIRDMAn ranking followed by centered log-ratio plotting, was performed for the parameters TEWL (Figures 2C, D; Figure 3B) and corneometer (Figures 2E, F; Figure 3C), leading to a reduced list of taxa associated with these measures as well. Taxa most associated with low TEWL included some typical of skin such as *Staphylococcus* and *Bacillus*, but nearly all were of relatively low prevalence. The taxa associated with high corneometer measures included four skin typical taxa: *Janibacter*, *Roseomonas*, *Sphingomonas*, and *Lactobacillus*. Surprisingly, *Cutibacterium*, while the most abundant genus in the dataset (and in the cheek microbiome as shown in previous studies), showed no significant relationship with age, only trended negatively with increasing GCFW (*R* = −0.31, *p* = 1.3e-08; *β* = −0.1, *p* = 0.18) (Supplementary Figure S2), and was not detected as one of the taxa showing strong association with the skin aging signs and quality parameters analyzed in this multi-study analysis.

## 4 Discussion

Conducting a multi-study analysis can be a powerful approach, allowing for the investigation of questions not able to be addressed by individual smaller studies. For microbiome studies, which gather large and valuable amounts of data, it allows for secondary analysis while bypassing the need to generate new expensive datasets (Agostinetto et al., 2022). It can also help to overcome the issue of low generalizability of results, as many studies tend to focus on just one population. Here we used a 3-step framework to address the link between the skin microbiome and three signs of aging: 1) deposit sequence data from 13 different studies into Qiita, a web-based platform designed to facilitate microbiome meta-analyses; 2) curate the corresponding metadata, improving the harmonization of the data across the studies and 3) use Qiita’s standardized bioinformatic pipeline for data processing and analysis. The present report shows that while data harmonization remains a challenge, it can be tackled in part by appropriate bioinformatic tools and methods.

Despite large study effects, we were able to assemble a multi-study analysis to reproduce patterns of skin microbiome variation consistent with previous results while also identifying new microbiome associations with skin quality and aging parameters. Consistent with much of the existing literature, our results showed that microbial diversity on cheek skin was higher in older individuals than in younger adults, and that *Cutibacterium* trended lower in older individuals (Kim et al., 2019; Huang et al., 2020; Larson et al., 2022). Additionally, we observed that microbial diversity tended to decrease with decreasing skin barrier function as measured by increasing TEWL. This result was consistent with an earlier report showing a similar correlation (Park et al., 2021).

Using models that can account for multiple known variables with appreciable effect size such as study and age (e.g., Huang et al., 2021), we were able to identify taxa that appear to be more related to an aging sign (GCFW) and skin quality parameters (TEWL, corneometer) than to chronological age. While previous studies have reported correlations between the skin microbiome and clinical parameters such as UV spots, dark spots and skin hydration (Kim et al., 2019), it is difficult to determine whether these patterns are due to the aging process *per se*, rather than to the physiological manifestations of aging. Additionally, prediction models that have been applied to hydration measures (corneometer) with good performance involved samples from the leg instead of the face (Carrieri et al., 2021). In the present analysis, we focused on samples from cheek skin, which is the key body site for apparent age. More recent studies are shedding additional light on associations of the skin microbiome, notably particular clades of *S. epidermidis* or *C. acnes*, with biophysical traits of skin aging, such as collagen quality and quantity (Zhou et al., 2023a; Xia et al., 2023). Our study adds to this nascent, but growing body of knowledge aiming to better understand the implications of the microbiome in skin aging, by identifying the changes associated with signs of skin aging, rather than chronological aging alone. It sets the path for new studies to further characterize and validate new microbiome biomarkers of skin aging signs.

We found that taxa that correlated with high GCFW include mainly environmental bacteria such as *Kaistella* and other taxa from the phylum Actinomycetes (*Brevibacterium* and *Microbacterium*). While some of these taxa may be found on healthy skin (Khayyira et al., 2020), a high abundance on the skin has previously been linked to skin alterations due to air pollution (Leung et al., 2020) and skin inflammation (Huang et al., 2021; Li et al., 2021). For example, *Microbacterium* were previously found on the skin of a population exposed to high levels of polycyclic aromatic hydrocarbon pollutants (Leung et al., 2020) and in the dermis of patients with toenail infection (Li et al., 2021). *Brevibacterium* was reported to be predominant in skin samples of psoriatic lesions (Stehlikova et al., 2019), senile pruritis subjects (Huang et al., 2021) and on skin of elderly bedridden subjects often presenting dry skin with possible localized infected wounds (Nagase et al., 2020). Overall, several of the bacteria associated with high GCFW in our study have previously been identified in the context of skin microbiome dysbiosis associated with several skin inflammatory conditions.

Our findings also revealed key commensal gram-positive bacteria associated with low GCFW, including taxa from the two major skin phyla Firmicutes and Actinobacteria. Examples include *Staphylococcus* (taxon classified as *Staphylococcus equorum*) as well as *Kocuria*, *Peptostreptococcus,* and *Lysobacter*, which have been shown to be both skin and environment-related taxa*.* These taxa have been previously reported as being associated with anti-inflammation and enhanced skin health and appearance (Lebeer et al., 2022; Zanchetta et al., 2022). One study investigating the microbiome composition of hyperpigmented skin reported a possible protective effect of *Kocuria* that was predominant in skin with less hyperpigmentation and was identified as the most discriminant bacteria of dark spots (Zanchetta et al., 2022). Notably, one taxon classified as *Lactobacillus* was associated with higher corneometer measures in our analysis (Figure 3C). Such lactic acid bacteria have been reported to have anti-inflammatory benefits and capabilities in enhancing skin health and appearance when used as probiotics (Im et al., 2016; Lebeer et al., 2022). For example, the topical application of *Lactobacillus* strains was shown to reduce inflammatory lesions related to acne, presumably through modulating the immune response (Lebeer et al., 2022). These taxa associated with lower GCFW and higher corneometer measures in our study may represent potential biomarker candidates of younger skin appearance. While we were surprised that *Cutibacterium*, the most abundant skin taxon in our dataset, was not among those differentially associated with the parameters investigated in our study, it might be because this taxon is associated with other skin aging parameters not included in our study such as dark spots (Shibagaki et al., 2017; Misra et al., 2021), sebum level (Shibagaki et al., 2017), collagen (Zhou et al., 2023a), or other types of face wrinkles.

Altogether, the work presented here provides for the first time specific microbial features associated with GCFW, skin barrier function as measured by TEWL, and skin hydration measured by corneometer, while considering chronological age as a potential confounder. As with any multi-study analysis where independent studies designed to answer specific questions are combined, our study was limited by study effects, the causes of which may include differences in participant recruitment, sample collection, storage, reagents, and sequencing protocol. However, the identification of trends consistent with existing literature despite study effects suggest that our results may be more generalizable to other studies than results reported in single studies alone. Another limitation was the use of 16S amplicon technology, which while providing an adequate representation of the skin microbial community, limits our ability to gain reliable species level resolution or functional insights. In our study, different members of the same genus (e.g., *Brevibacterium, Staphylococcus, Corynebacterium*) were found as associated with opposing measures, for example, both high and low GCFW, suggesting differential association at the species or strain levels. Strain or isolate-dependent effects have been reported in other studies, such as for *Staphylococcus epidermidis* and *Cutibacterium acnes* in a frailty study in older adults (Larson et al., 2022), or for *S. epidermidis’* effect on the structure of skin cells (Landemaine et al., 2023). Therefore, further studies using other omics and importantly, experimental research (*in vitro*, *ex vivo*, and *in vivo*), are needed to validate our findings. For example, metabolomics could be used to identify the metabolites associated with aging-related microbial biomarkers, offering potential product opportunities to rebalance the skin microbiome for healthier looking skin. Alternatively, meta-transcriptomics could offer insight into the genes expressed in aging-related microbes, offering targets for genetic engineering in other commensal strains. Finally, our analysis focused on studies of the microbiome on the skin surface layer as these are the most common due to ease of non-invasive sampling. However, it may be important for future studies to consider how microbes may be involved in aging of the lower skin layers, such as impacts on collagen production by fibroblasts of the dermis, especially as previous studies have shown that there are microbial interactions that occur in the lower layers of the skin either directly (Nakatsuji et al., 2013) or through metabolites (Yu et al., 2019). Our study provides the foundation and framework for these types of future clinical and *in vitro* research to identify new microbiome biomarkers of apparent skin age and to develop new skincare solutions promoting higher skin quality during aging.

## Data Availability

The datasets presented in this study can be found in online repositories. The names of the repository/repositories and accession number(s) can be found below: all the info is or will be contained in the Supplementary Table S1.

## Supplementary material

SUPPLEMENTARY TABLE S1 Detailed information about the 13 studies.

SUPPLEMENTARY TABLE S2 Top taxa associated with Grade of Wrinkles.

SUPPLEMENTARY TABLE S3 Top taxa associated with TEWL.

SUPPLEMENTARY TABLE S4 Top taxa associated with Corneometer.

SUPPLEMENTARY FIGURE S1 Distribution of values for **(A)** age, **(B)** grade of wrinkles, **(C)** TEWL, and **(D)** corneometer across studies.

SUPPLEMENTARY FIGURE S2
*Cutibacterium* correlations with grade of wrinkles and age. Scatterplots show the centered log ratio (CLR) of all taxa assigned to the genus *Cutibacterium*, with increasing grade of wrinkles **(A)** and age **(B)**. Show in the inset are the Spearman correlation values (R, p) for the full dataset, along with the statistics for a mixed effects model [Est (β), p] using study as a random effect. Lines show a fitted regression line, and shaded areas show the 95% CI. Point colors represent study. Only studies that had data on both age and grade of wrinkles were utilized.

SUPPLEMENTARY FIGURE S3 Plots showing the centered-log ratio (CLR) abundance for the top 22 taxa associated with higher grade of wrinkles versus grade of wrinkles.

SUPPLEMENTARY FIGURE S4 Plots showing the centered-log ratio (CLR) abundance for the top 22 taxa associated with higher grade of wrinkles versus age.

SUPPLEMENTARY FIGURE S5 Plots showing the centered-log ratio (CLR) abundance for the top 13 taxa associated with lower grade of wrinkles versus grade of wrinkles.

SUPPLEMENTARY FIGURE S6 Plots showing the centered-log ratio (CLR) abundance for the top 13 taxa associated with lower grade of wrinkles versus host age.
