# Supplementary figures and images for: A multi-study analysis enables identification of potential microbial features associated with skin aging signs
Source: Front Aging. 2024 Jan 11;4:1304705. doi: 10.3389/fragi.2023.1304705 (PMC10868648; doi:10.3389/fragi.2023.1304705)

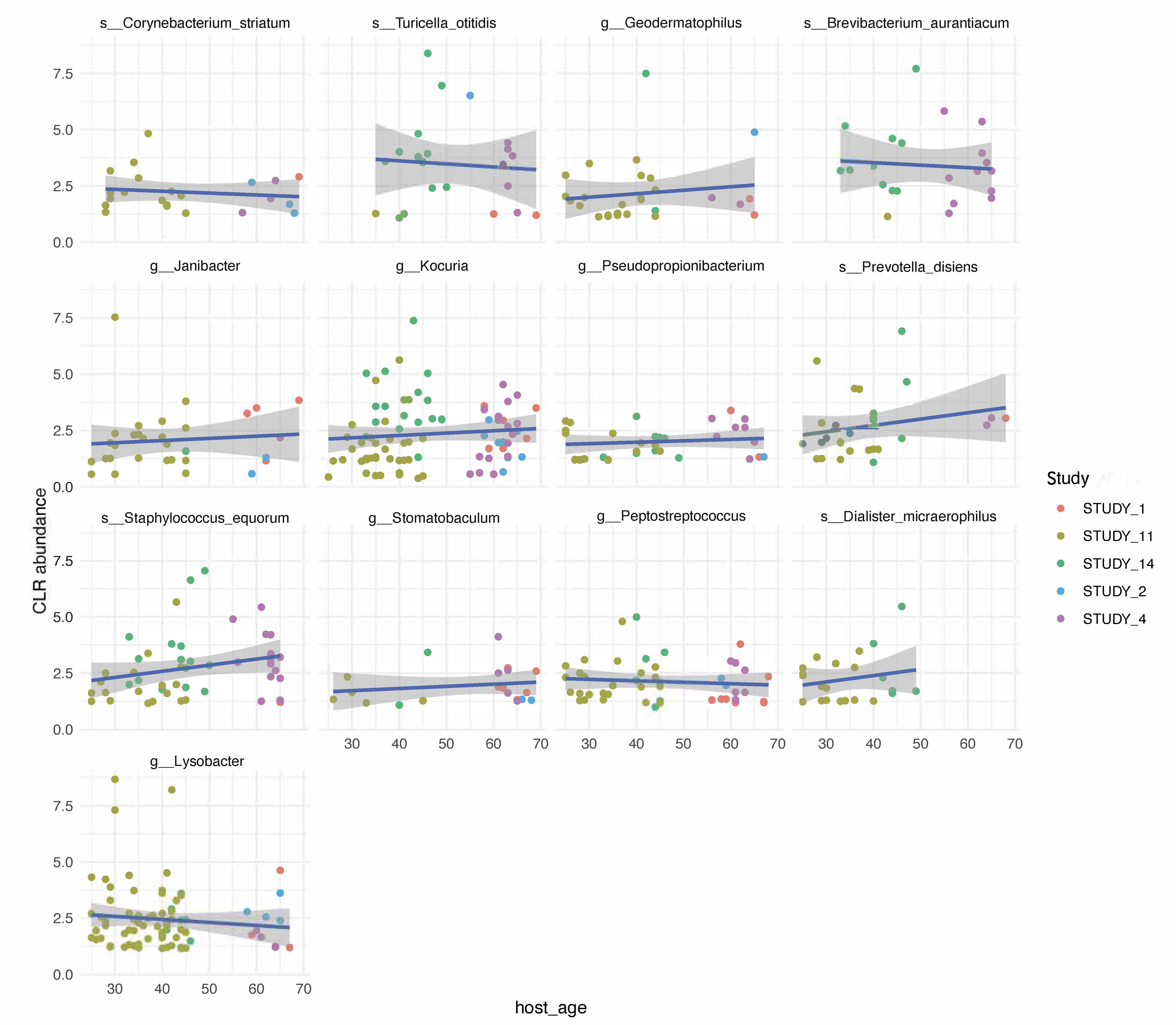

Supplement: Supplementary file 1 [file Image6.tif]

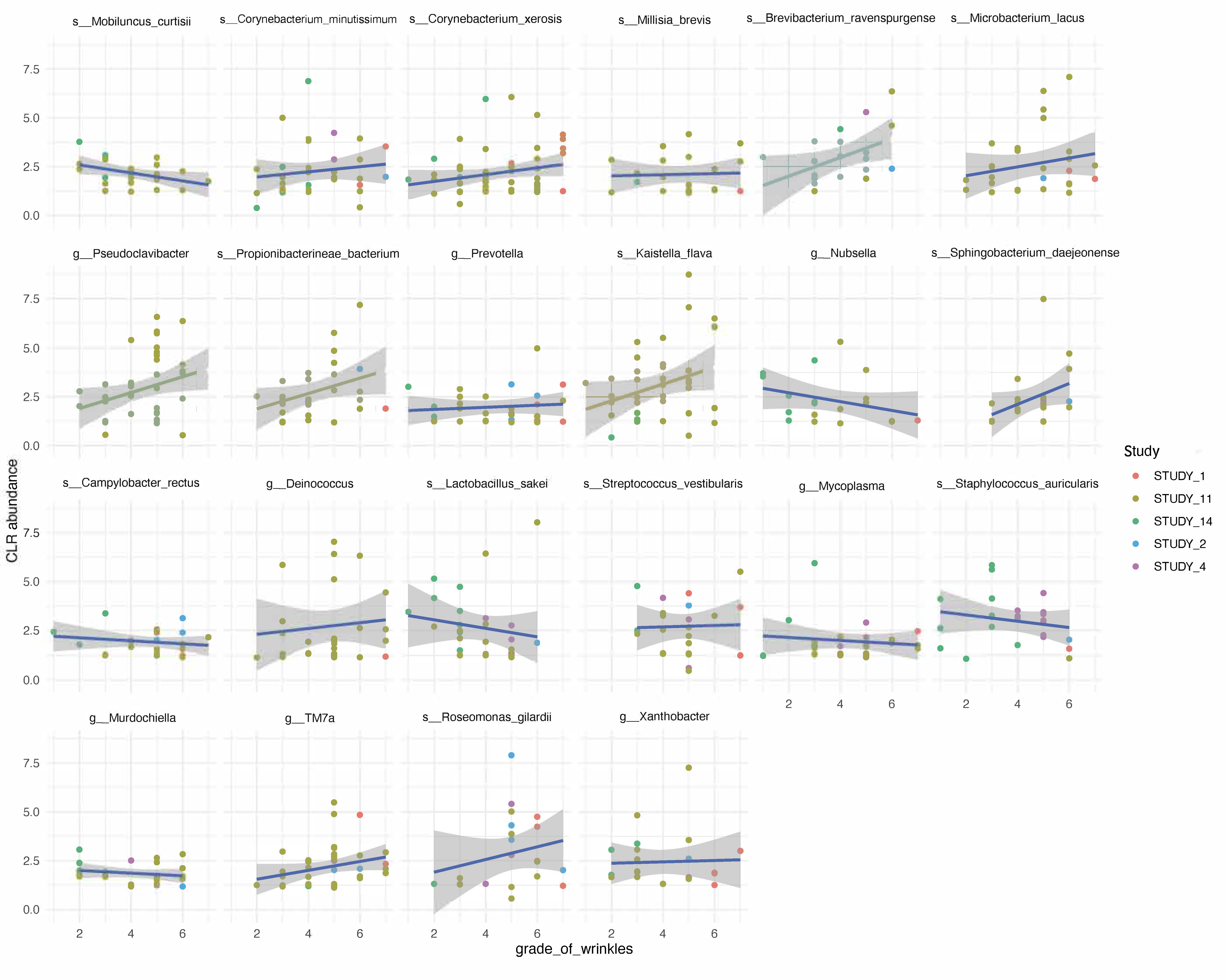

Supplement: Supplementary file 2 [file Image3.tif]

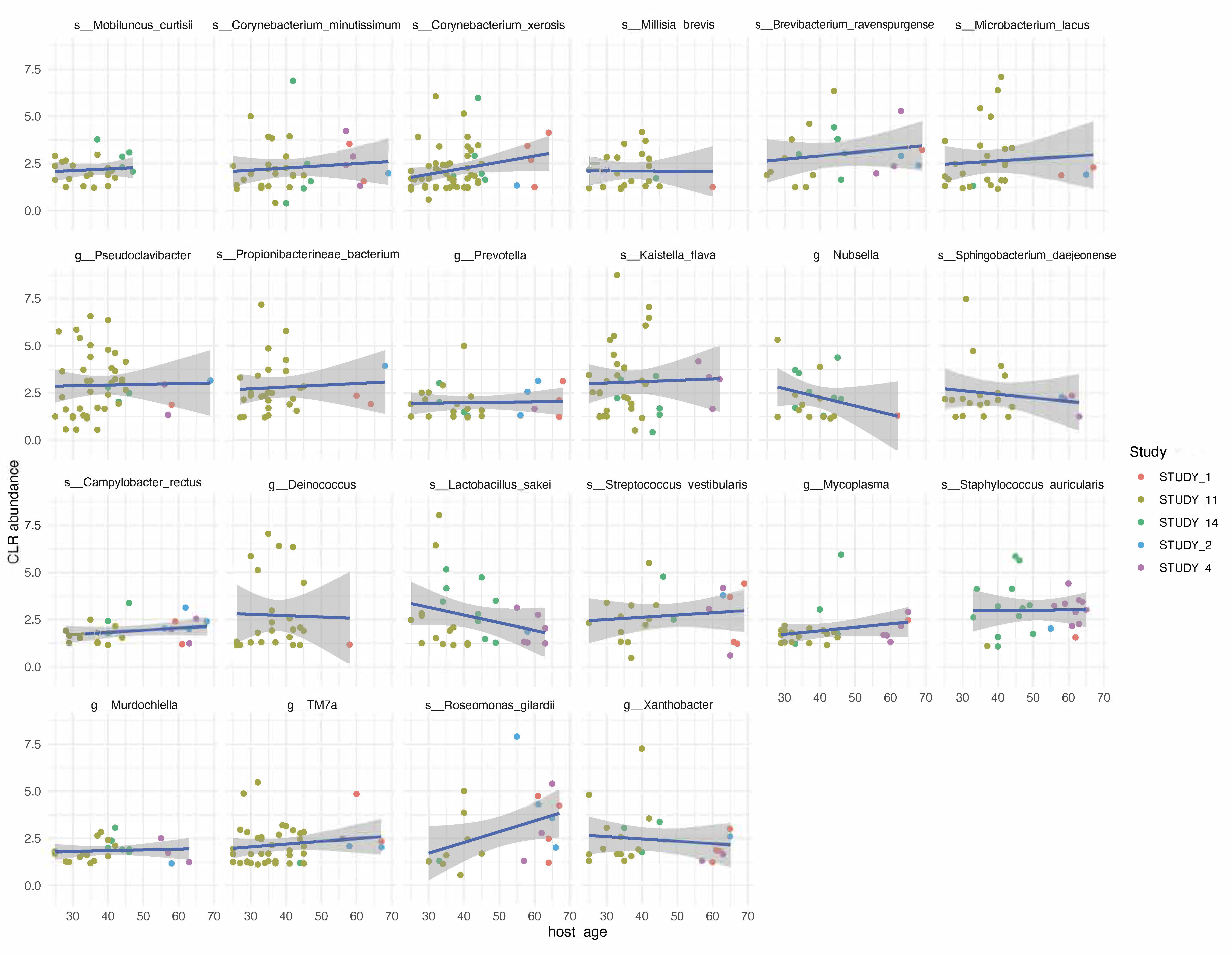

Supplement: Supplementary file 3 [file Image4.tif]

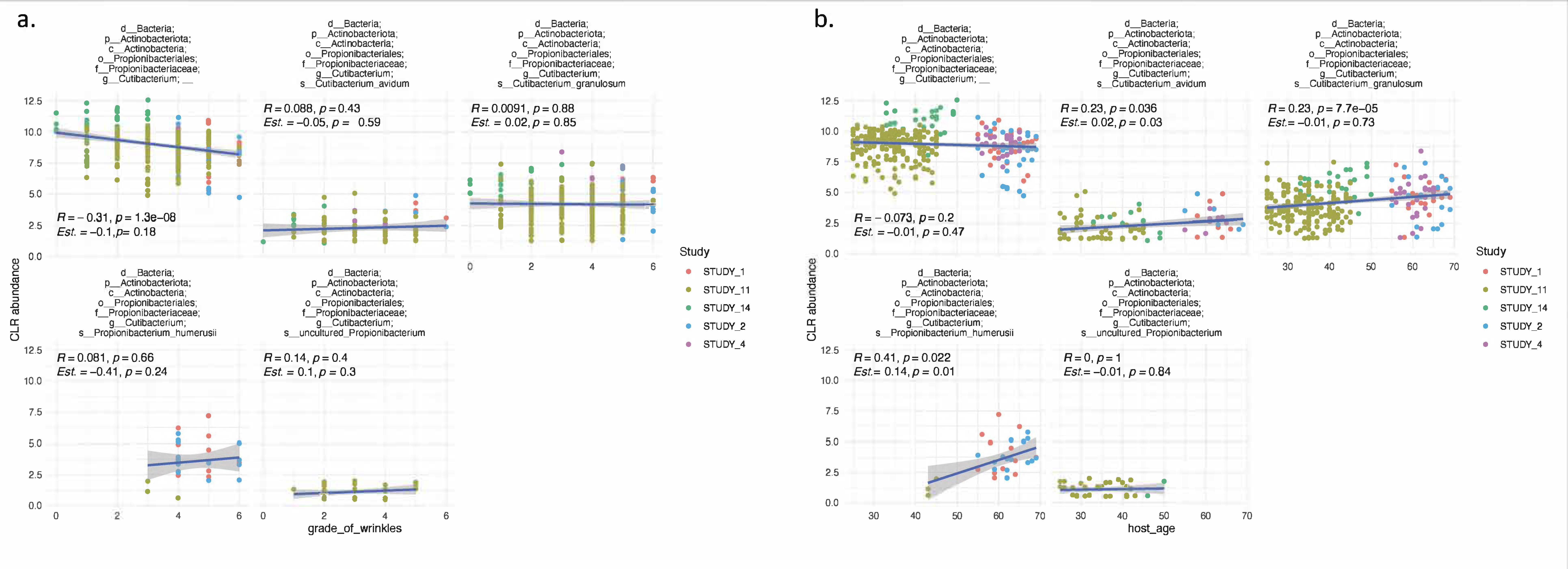

Supplement: Supplementary file 4 [file Image2.tif]

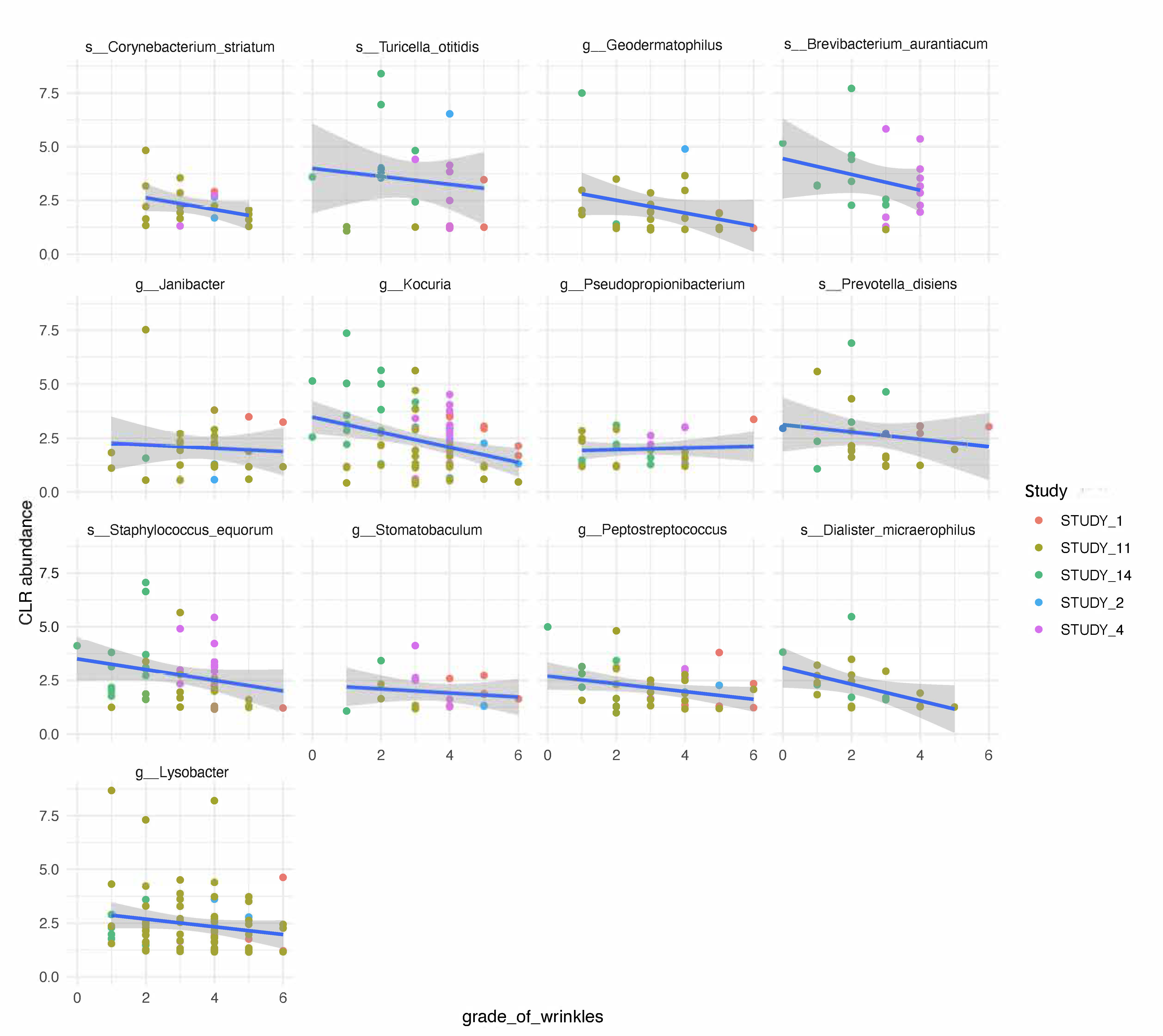

Supplement: Supplementary file 7 [file Image5.tif]
